# Impact of time between diagnosis to treatment in Acute Type A Aortic Dissection

**DOI:** 10.1038/s41598-021-83180-6

**Published:** 2021-02-10

**Authors:** Caleb R. Matthews, Mackenzie Madison, Lava R. Timsina, Niharika Namburi, Zainab Faiza, Lawrence S. Lee

**Affiliations:** 1 Division of Cardiothoracic Surgery, Indiana University School of Medicine, 545 Barnhill Drive, Indianapolis, IN 46202 USA; 2 Indiana University Health Methodist Hospital, 1801 N. Senate Blvd., Suite 3300, Indianapolis, IN 46202 USA

**Keywords:** Cardiology, Health care, Medical research

## Abstract

There is a paucity of data describing the effect of time interval between diagnosis and surgery for Acute Type A Aortic Dissection. We describe our 8-year experience and investigate the impact of time interval between symptom onset, diagnosis and surgery on outcomes. Retrospective single-center study utilizing our Society of Thoracic Surgeons registry and patient records. Subjects were grouped by time interval between radiographic diagnosis and surgical treatment: Group A (0–4 h), Group B (4.1–8 h), Group C (8.1–12 h), and Group D (12.1 + h). Data were analyzed to identify factors associated with mortality and outcomes. 164 patients were included. Overall mortality was 21.3%. Group C had the greatest intervals between symptom onset to diagnosis to surgery, and also the highest mortality (66.7%). Preoperative tamponade, cardiac arrest, malperfusion, elevated creatinine, cardiopulmonary bypass time, and blood transfusions were associated with increased mortality, while distance of referring hospital was not. Time intervals between symptom onset, diagnosis and surgery have a significant effect on mortality. Surgery performed 8–12 h after diagnosis carries the highest mortality, which may be exacerbated by longer interval since symptom onset. Time-dependent effects should be considered when determining optimal strategy especially if inter-facility transfer is necessary.

## Introduction

Acute Type A Aortic Dissection (ATAAD) remains a highly morbid diagnosis with relatively high mortality and few definitive treatment options aside from surgery^1–3^. Mortality increases in a time-dependent manner, with published data demonstrating mortality increases of 1–2% per hour in the first 24 h; thus, early diagnosis and treatment are paramount in achieving successful outcomes^1,4–7^. Overall incidence of ATAAD is difficult to ascertain as many patients die before reaching medical care. Populations studies have estimated overall incidence of approximately 11.9 cases per 100,000 people per year^2^. Emergency surgery is the preferred management strategy once the diagnosis of ATAAD is confirmed^3,8–10^. While surgical intervention historically carried all-cause in-hospital mortality rates of over 40%, recent studies have demonstrated improving survival presumably as a result of earlier diagnosis and improved surgical technique^3,7,11–13^.

The International Registry for Acute Aortic Disease (IRAD) and others have shown that preoperative risk factors associated with poor prognosis include age greater than 70 years, male sex, cardiogenic shock, kidney failure, history of atherosclerosis, stroke and malperfusion syndrome (MPS)^3,8,9,11^. However, despite the crucial importance of expeditious surgical intervention for ATAAD, there are few reports investigating the effect of time interval between diagnosis and surgery on outcomes. Studies have documented potential delay in treatment either due to misdiagnosis or delayed diagnosis, often related to low clinical index of suspicion in the Emergency Department setting^25,26^. Regardless of reason for delay, whether occurring prior to presentation or during and after diagnostic evaluation, there is relatively limited literature on the role these interval delays have on outcomes.

We report our recent experience with ATAAD with a focus on the effects of time between symptom onset to diagnosis to treatment on clinical outcomes. We hypothesized that increasing time interval between diagnosis and surgery would lead to greater adverse outcomes, and that patients requiring longer distances for inter-facility transfer would have longer time interval to treatment and consequently greater mortality.

## Materials and methods

This single-center retrospective study was approved by the Institutional Review Board of Indiana University and conducted in accordance with all University guidelines and regulations. Informed consent by individual study patients was waived by the Institutional Review Board given the retrospective nature of the study. Our institutional Society of Thoracic Surgeons data registry was queried to identify all patients who underwent surgery for ATAAD repair at our institution between 2009 and 2016. Preoperative, intraoperative, and postoperative variables were extracted, and individual patient charts were reviewed for supplemental data collection. Patients with prior aortic dissection or incomplete medical documentation were excluded.

Symptom onset time was defined by each patient’s subjective reports as recorded in the medical record. Presentation time was defined by the time stamp of entry into the Emergency Department (ED). Diagnosis time was defined by the time stamp on the computed tomography (CT) study where ATAAD was identified. Treatment start time was defined as the time of entry into the operating room (OR). The interval between diagnosis time and treatment start time was then calculated and defined as the Time to Intervention (TtI). We divided the study cohort into groups based on the TtI: 0–4 h (Group A), 4.1–8 h (Group B), 8.1–12 h (Group C), and 12.1 +  h (Group D). Baseline characteristics, operative variables, and postoperative outcomes were compared across susbgroups. The facility to which each patient had initially presented was also identified, and the distance from that facility to our institution was calculated using standard online mapping applications.

### Statistical analysis

Descriptive analysis was performed using proportions from frequency distribution for categorical variables and mean (SD) or median for continuous variables to describe the patient characteristics in the study cohort. In bivariate analyses of continuous variables, we performed Kruskal–Wallis tests of the hypotheses as a non-parametric version of testing differences across groups. For categorical variables, Fisher’s exact tests were performed. Multivariable analyses were performed to examine the relationship between short and long-term outcomes after accounting for the confounding effect of demographic variables (age, gender, race), comorbidities, conditions at presentation, and operative variables. Multivariable maximum likelihood logistic regression was used to analyze binary outcomes. To account for the rarity of some outcomes, penalized logistic regressions were utilized. Multivariable poisson regression and linear regression were performed for count outcome data and for continuous outcome data, respectively. Multicollinarity in all models was assessed using variance inflation factor (VIF), and any variables with VIF > 10 were considered to be collinear and hence were removed from the final multivariable models. All analyses were completed using Stata/SE 14.2 and the hypotheses tested at 0.05 level of significance.

## Results

178 patients presenting with ATAAD were identified. Of these, 14 were excluded because of insufficient preoperative imaging data availability. 164 patients comprised the final study cohort: 104 in Group A, 28 in Group B, 6 in Group C, and 26 in Group D. Mean age was 57.3 years, with 64.6% male and 78.1% Caucasian (Table 1). Baseline characteristics and presenting signs amongst the subgroups were similar except that Group D had more chronic kidney disease, higher rates of dialysis, and lower starting hematocrit (Table 1). All patients had chest or back pain as a presenting symptom. Preoperative risk factors associated with increased mortality included cardiac arrest, pericardial tamponade, MPS (specifically, mesenteric malperfusion), and elevated creatinine. Group C had the longest time interval between symptom onset and presentation (14.0 h) and between symptom onset and diagnosis (26.4 h) (p = 0.0001). Group D had the longest interval between symptom onset and intervention (54.5 h) (Table 1).

**Table 1:** Patient characteristics by survival status and by group.

Characteristics	Alive (n = 129)	Dead (n = 35)	p-value	Group A 0–4 h (n = 104)	Group B 4.1–8 h (n = 28)	Group C 8.1–12 h (n = 6)	Group D 12.1 + h (n = 26)	p-value
Age^a^, years	56.8 ± 14.1	59.4 ± 13.9	0.3869	57.4 ± 13.8	56.1 ± 15.2	58.0 ± 11.5	58.2 ± 15.0	0.9045
Body Mass Index^a^	29.4 ± 5.8	30.4 ± 4.7	0.2688	29.71 ± 6.0	31.2 ± 4.6	30.4 ± 5.6	27.6 ± 4.4	0.1031
Gender			0.232					0.689
Male	80 (62.0)	26 (74.3)		66 (63.5)	18 (64.3)	3 (50.0)	19 (73.1)	
Female	4 (38.0)	9 (25.7)		38 (36.5)	10 (35.7)	3 (50.0)	7 (26.9)	
Race			0.068					0.194
White	105 (81.4)	23 (65.7)		84 (80.8)	22 (78.6)	6 (100.0)	16 (61.5)	
Black	17 (13.2)	10 (28.6)		16 (15.4)	3 (10.7)	0	8 (30.8)	
Other	7 (5.4)	2 (5.7)		4 (3.8)	3 (10.7)	0	2 (7.7)	
Tobacco use	62 (48.1)	13 (37.1)	0.339	41 (39.4)	14 (50.0)	3 (50.0)	17 (65.4)	0.108
Hypertension	51 (39.5)	13 (37.1)	0.847	39 (37.5)	1 (42.9)	4 (66.7)	9 (34.6)	0.487
Hyperlipidemia	13 (10.1)	2 (5.7)	0.338	9 (8.7)	2 (7.4)	1 (16.7)	3 (11.5)	0.679
COPD	11 (8.5)	5 (14.3)	0.235	8 (7.7)	2 (7.4)	1 (16.7)	5 (19.2)	0.245
CKD	6 (4.7)	5 (14.3)	0.058	3 (2.9)	1 (3.6)	0	7 (26.9)	0.001
Dialysis	2 (1.6)	2 (5.7)	0.200	1 (1.0)	0	0	3 (11.5)	0.047
Diabetes	15 (11.6)	4 (11.4)	0.620	10 (9.6)	6 (21.4)	2 (33.3)	1 (3.9)	0.055
CAD	12 (9.3)	5 (14.3)	0.282	8 (7.7)	6 (21.4)	0	3 (11.5)	0.201
Liver disease	1 (0.8)	0	> 0.999	0	1 (3.6)	0	0	0.366
Marfan’s	2 (1.6)	1 (2.9)	0.516	2 (1.9)	1 (3.6)	0	0	0.748
Loeys-Dietz	1 (0.8)	0	> 0.999	0	0	0	1 (3.9)	0.195
Stroke/Transient Ischemic Attack	11 (8.5)	7 (20.0)	0.068	8 (7.7)	4 (14.3)	2 (33.3)	4 (15.4)	0.117
Prior sternotomy	6 (4.7)	3 (8.6)	0.404	4 (3.9)	0	0	5 (19.2)	0.022
**Presentation**								
Tamponade	5 (3.9)	5 (14.3)	0.037	8 (7.7)	1 (3.6)	0	1 (3.9)	0.863
Myocardial Infarction	5 (3.9)	1 (2.9)	> 0.999	4 (3.9)	0	1 (16.7)	1 (3.9)	0.266
Aortic Insufficiency	21 (16.3)	3 (8.6)	0.417	19 (18.3)	3 (10.7)	0	2 (7.7)	0.442
Cardiogenic Shock	5 (3.9)	4 (11.4)	0.098	8 (7.7)	1 (3.6)	0	0	0.623
Cardiac Arrest	0	2 (5.7)	0.045	1 (1.0)	1 (3.6)	0	0	0.599
Malperfusion	12 (9.3)	8 (22.9)	0.041	11 (10.6)	4 (14.3)	2 (33.3)	3 (11.5)	0.340
Upper Extremity	2 (1.6)	0	> 0.999	1 (1.0)	0	0	1 (3.9)	0.381
Lower Extremity	10 (7.8)	6 (17.1)	0.112	9 (8.7)	4 (14.3)	1 (16.7)	2 (7.7)	0.535
Mesenteric	2 (1.6)	5 (14.3)	0.005	3 (2.9)	2 (7.4)	1 (16.7)	1 (3.9)	0.191
Cerebral	1 (0.8)	0	> 0.999	1 (1.0)	0	0	0	> 0.999
Creatinine^a^, mg/dL	1.2 ± 0.8	1.8 ± 1.7	0.0001	1.2 ± 1.0	1.1 ± 0.4	1.4 ± 0.8	1.8 ± 1.8	0.7195
Hematocrit^a^, %	38.0 ± 6.0	37.8 ± 6.9	0.9301	38.7 ± 5.0	39.8 ± 6.1	39.3 ± 4.1	32.7 ± 8.1	0.001
**Symptom onset interval**								
Symptom Onset to Presentation^b^, hours			3.2 (4.4)	4.2 (4.6)	14.0 (19.9)	11.0 (64.1)	0.0001	
Symptom Onset to Diagnosis^b^, hours			3.2 (5.0)	6.8 (7.8)	26.4 (64.6)	12.1 (49.8)	0.0001	
Symptom Onset to Intervention^b^, hours			3.9 (4.6)	8.3 (6.9)	33.8 (58.5)	54.5 (118.2)	0.0001	

Values are expressed as number (%) unless otherwise indicated. a, mean ± standard deviation; b, median (interquartile range); COPD, chronic obstructive pulmonary disease; CKD, chronic kidney disease; CAD, coronary artery disease.

Intraoperative characteristics are listed in Table 2. All patients underwent ascending aorta replacement with prosthetic tube graft reconstruction. In addition, 63% underwent concomitant hemiarch replacement, 38% underwent aortic root replacement (including valve-sparing), and 16% underwent total arch replacement. Arterial cannulation was achieved most commonly via the femoral route (74%) followed by direct aortic (26%) and axillary approaches (14%). Longer CPB time and greater intraoperative blood product transfusion requirement were both associated with increased mortality, but other intraoperative variables including cannulation strategy, extent of repair, aortic valve intervention, coronary artery bypass grafting, and extracorporeal membrane oxygenation (ECMO) requirement were not.

**Table 2:** Intraoperative variables by survival status and by group.

Variable	Alive (n = 129)	Dead (n = 35)	p-value	Group A 0–4 h (n = 104)	Group B 4.1–8 h (n = 28)	Group C 8.1–12 h (n = 6)	Group D 12.1 + h (n = 26)	p-value
**Procedure**								
Hemi-arch	80 (62.0)	2 (65.7)	0.688	65 (62.5)	17 (60.7)	6 (100.0)	15 (57.7)	0.272
Total arch	19 (14.7)	8 (22.9)	0.303	14 (13.5)	8 (28.6)	1 (16.7)	4 (15.4)	0.275
Root replacement	36 (27.9)	11 (31.4)	0.683	32 (30.8)	7 (25.0)	3 (50.0)	5 (19.2)	0.420
Valve sparing root	11 (8.5)	5 (14.3)	0.338	9 (8.7)	3 (10.7)	1 (16.7)	3 (11.5)	0.676
Arch branch bypass	55 (42.6)	16 (45.7)	0.744	44 (42.3)	14 (50.0)	3 (50.0)	10 (38.5)	0.813
AV replacement	28 (21.7)	7 (20.0)	> 0.999	24 (23.1)	5 (17.9)	2 (33.3)	4 (15.4)	0.671
AV repair	5 (3.9)	1 (2.9)	0.776	4 (3.9)	1 (3.6)	0	1 (3.9)	> 0.999
Coronary reconstruction	30 (23.3)	8 (22.9)	> 0.999	25 (24.0)	3 (10.7)	4 (66.7)	6 (23.1)	0.038
CABG	9 (7.0)	5 (14.3)	0.180	8 (7.7)	1 (3.6)	2 (33.3)	3 (11.5)	0.112
Mitral valve replacement	1 (0.8)	0	> 0.999	0	0	0	1 (3.9)	0.195
ECMO	9 (7.0)	3 (8.6)	0.720	5 (4.8)	5 (17.9)	1 (16.7)	1 (3.9)	0.067
**Cannulation strategy**								
*Arterial*								
Aortic	33 (25.6)	9 (25.7)	> 0.999	33 (31.7)	2 (7.4)	1 (16.7)	6 (23.1)	0.042
Axillary	16 (12.4)	7 (20.0)	0.275	10 (9.6)	7 (25.0)	2 (33.3)	4 (15.4)	0.069
Femoral	96 (74.4)	25 (71.4)	0.721	78 (75.0)	20 (71.4)	5 (83.3)	18 (69.2)	0.879
Innominate	12 (9.3)	2 (5.7)	0.736	9 (8.7)	2 (7.4)	0	3 (11.5)	0.907
*Venous*								
Caval/bicaval	9 (7.0)	4 (11.4)	0.478	6 (5.8)	3 (10.7)	2 (33.3)	2 (7.7)	0.101
Femoral	8 (6.2)	3 (8.6)	0.703	4 (3.9)	1 (3.6)	0	6 (23.1)	0.014
Right atrial	112 (86.8)	29 (82.9)	0.585	94 (90.4)	25 (89.3)	4 (66.7)	18 (69.2)	0.019
**Intraoperative details**								
CPB time^a^, minutes	244.2 ± 82.2	294.6 ± 163.8	0.0132	246.1 ± 102.7	258.3 ± 108.8	354.9 ± 240.0	264.4 ± 78.3	0.2304
Cross clamp time^a^, minutes	136.2 ± 75.0	139.2 ± 77.4	0.8658	132.2 ± 78.3	156.0 ± 60.0	132.3 ± 96.6	138.2 ± 72.0	0.3889
Circulatory arrest time^a^, minutes	24.0 ± 17.4	24.6 ± 18.6	0.7722	24.4 ± 18.0	24.4 ± 18.3	36.6 ± 36.6	18.0 ± 18.0	0.3764
Blood products transfused^a^	8.2 ± 6.1	14.9 ± 10.1	< 0.00001	9.3 ± 7.4	10.3 ± 5.1	17.3 ± 16.9	8.7 ± 7.4	0.2968

Values are expressed as number (%) unless otherwise indicated. a, mean ± standard deviation; AV, aortic valve; CABG, coronary artery bypass grafting; ECMO, extracorporeal membrane oxygenation; CPB, cardiopulmonary bypass.

Median postoperative follow-up was 556.6 days. Overall and 30-day mortality for the entire cohort was 21.3% and 18.3%, respectively. Across subgroups, overall mortality was significantly different but 30-day mortality was not (Table 3): overall mortality for Groups A, B, C, and D was 20.1%, 25.0%, 66.7%, and 11.6%, respectively (p = 0.040), and 30-day mortality was 16.4%, 25.0%, 50.0%, and 11.6%, respectively (p = 0.121). Group C had the greatest interval between symptom onset and presentation. In-hospital postoperative outcomes were similar across subgroups. Postoperative renal failure requiring dialysis was strongly associated with mortality risk, occurring in 38% of patients who died versus 6% of those who survived. Postoperative pneumonia was most common in Group A, occurring in 27% of patients. Aortic re-intervention rates at any point during the follow-up period for Groups A, B, C, and D were 20.0%, 14.3%, 16.7%, and 15.4%, respectively. Table 4 lists the intra- and post-operative pathologies most commonly present in those that died. Multivariate analysis demonstrated that Group C was likely an independent predictor of overall mortality (Odds Ratio 12.76 [0.86–190.41], p = 0.065), while Group D was a predictor of overall survival (Odds Ratio 0.06 [0.01–0.79], p = 0.032).

**Table 3:** Postoperative outcomes by survival status and by group.

Variable	Alive (n = 129)	Dead (n = 35)	p-value	Group A 0–4 h (n = 104)	Group B 4.1–8 h (n = 28)	Group C 8.1–12 h (n = 6)	Group D 12.1 + hours (n = 26)	p-value
Overall mortality	–	–		21 (20.2)	7 (25.0)	4 (66.7)	3 (11.5)	0.040
30-day mortality	–	–		17 (16.4)	7 (25.0)	3 (50.0)	3 (11.5)	0.121
Length of stay^a^, days	13.4 ± 9.7	5.4 ± 5.9	< 0.00001	11.2 ± 9.1	9.3 ± 7.2	11.3 ± 18.2	16.4 ± 10.3	0.0042
Permanent stroke	17 (13.2)	6 (17.1)	0.576	16 (15.5)	1 (3.6)	0	6 (23.1)	0.162
Paralysis	3 (2.3)	3 (8.6)	0.112	5 (4.8)	0	0	1 (3.9)	0.760
Renal failure	11 (8.5)	11 (31.4)	0.001	14 (13.5)	4 (14.3)	2 (33.3)	2 (7.7)	0.275
Dialysis required	8 (6.2)	12 (34.3)	< 0.00001	11 (10.6)	4 (14.3)	2 (33.3)	3 (11.5)	0.340
Ventilator time^a^, hours	49.2 ± 73.0	69.1 ± 82.8	0.1653	52.8 ± 59.5	38.2 ± 41.5	139.9 ± 284.4	52.6 ± 40.6	0.6073
Reintubation	19 (14.7)	6 (17.1)	0.789	16 (15.5)	2 (7.4)	1 (16.7)	6 (23.1)	0.385
ICU time, hours	103.3 (97.1)	70.5 (33.5)	0.0548	102.2 (104.0)	88.8 (54.4)	113.5 (79.4)	77.8 (40.7)	0.9064
Pneumonia	30 (23.3)	2 (5.7)	0.028	28 (26.9)	1 (3.6)	0	3 (11.5)	0.013
Re-operation for bleed	4 (3.1)	6 (17.1)	0.007	5 (4.8)	2 (7.4)	0	3 (11.5)	0.555
30-day re-admission	50 (38.8)	4 (11.4)	0.002	38 (36.5)	6 (21.4)	1 (16.7)	9 (34.6)	0.445
Re-do aortic surgery^b^	27 (20.9)	2 (5.7)	0.044	20 (19.2)	4 (14.3)	1 (16.7)	4 (15.4)	0.945

Values are expressed as number (%) unless otherwise indicated. a, mean ± standard deviation; ICU, intensive care unit. b, indicates need for re-do aortic surgery at any point during the follow-up period.

**Table 4:** Comorbid pathology present in patients that died.

Pathology	Frequency (%)
Cardiac arrest	33.0
Cardiogenic shock	30.8
Multi-system organ failure	28.0
Renal failure	23.1
Malperfusion syndrome	22.9
Acute myocardial infarction	8.0
Anoxic brain injury	7.7
Inability to wean cardiopulmonary bypass	5.1
Mediastinitis	2.3

Forty-five patients presented to our facility’s emergency department directly, while the remainder presented to an outside facility and were transferred to our institution. The study cohort represented patients who were transferred to our institution from 47 different facilities around the state (Table 5). The inter-facility transfer distance ranged from less than one mile up to 200 miles. 70% of transferring facilities were located within a 100-mile radius and comprised 85% of the study patients. The inter-facility transfer distance was not associated with increased TtI or mortality.

**Table 5:** Subjects per group by distance of transferring hospital.

Transfer distance from study institution	Number of hospitals	Number of patients	Group A 0–4 h	Group B 4.1–8 h	Group C 8.1–12 h	Group D 12.1 + h
< 25 miles	13	38	25	3	1	9
26–50 miles	5	9	6	1	0	2
51–100 miles	13	48	31	8	2	7
101–200 miles	14	24	7	14	0	3

## Discussion

The natural pathophysiology of ATAAD results in progressively increasing risk of complications including aortic rupture, tamponade, end-organ malperfusion, and acute heart failure secondary to aortic valve regurgitation or coronary ischemia. Historic studies demonstrated a time-dependent increase in mortality, which led to the widely accepted practice of expediting emergent surgery as definitive treatment^6^. Nonetheless, numerous studies have shown that delays or errors in diagnosis continue to persist with relative frequency: one study reported the median time between a patient’s arrival to the ED and diagnosis of ATAAD was 4.3 h, while others reported that delays in diagnosis occur in up to 25% of cases^7,10,14–16,24,25,26^.

Evangelista and colleagues analyzed 20-year IRAD data and reported an overall surgical mortality from ATAAD repair of 18%, while Lee and associates examined the STS registry and found operative mortality to be 17%^7,17^. Our 30-day and overall mortality rates are similar to these. Two of the most commonly cited pre-operative factors that portend worse prognosis are the presence of MPS and renal dysfunction^7,9,11,18^. Berretta et al*.* examined IRAD data and showed that while all forms of MPS were associated with increased mortality, mesenteric ischemia was particularly devastating and carried a 2.5-fold increase in mortality (63% vs. 24%)^19^. Our data show equally distressing outcomes with 83% of cases presenting with mesenteric ischemia resulting in death. Fan and colleagues reported that patients with preoperative renal dysfunction had higher postoperative mortality, while IRAD registry analysis by Mehta and associates showed preoperative renal dysfunction having an odds ratio of 4.77 for mortality^11,18^. Although preoperative CKD class was not associated with mortality in our cohort, we did find that an elevated preoperative creatinine level was associated with increased mortality. We initially hypothesized that more complex and longer operations, such as total arch or root replacement, would be associated with worse outcomes. Interestingly, the extent of operation did not significantly affect postoperative outcomes. Other studies also report the absence of relationship between intraoperative technical variables and outcomes^15,20^. We surmise that these findings suggest that pre-operative clinical status, rather than operative complexity, is the primary factor affecting postoperative mortality and outcomes.

**Figure 1:**
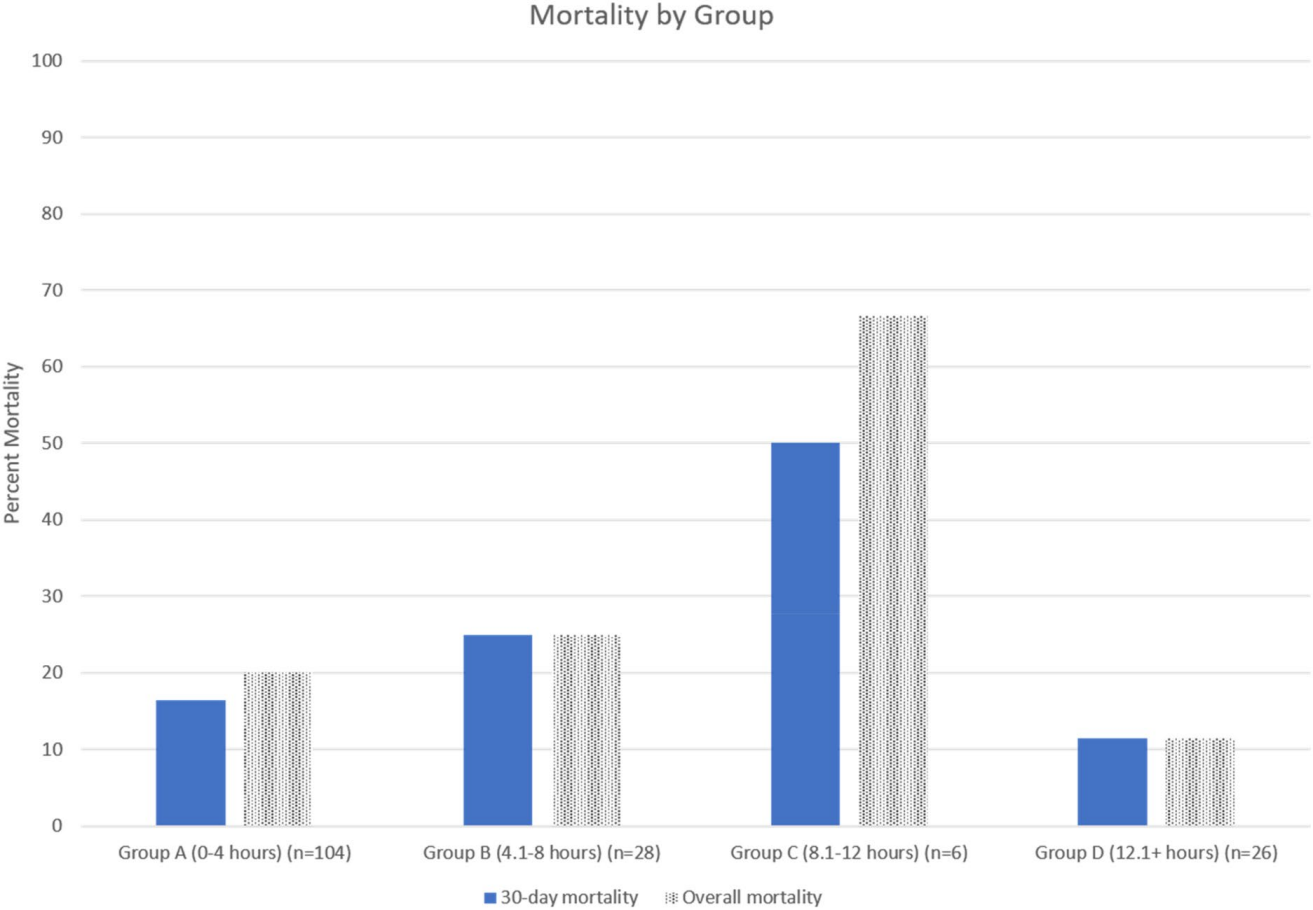
30-Day and overall mortality by group.

Our 30-day and overall mortality data follow an almost bell-curve like shape with respect to time between diagnosis and treatment (Fig. 1). Mortality was lowest for patients receiving surgery early (Group A) or late (Group D), and increased substantially for those receiving treatment between 4 and 12 h after diagnosis (Groups B and C). There were no differences in pre-operative characteristics or intraoperative techniques amongst these groups. The interval between symptom onset and presentation seems to be a key factor in affecting mortality, more so than the interval between symptom onset and intervention: Group C had the longest interval between symptom onset and presentation and also the highest mortality; on the other hand, Group D had a much shorter interval between symptom onset and presentation but a much longer interval to intervention, with ultimately significantly lower mortality. One explanation could be that patients who wait longer after symptom onset before presenting for evaluation are not receiving any medical treatment for the ATAAD during that interval and likely experiencing ongoing and progressive sequelae of the dissection. Quicker presentation to the ED results in earlier medical intervention including anti-impulse therapy, which may contribute to improved postoperative outcomes.

Group D had a relatively short interval between symptom onset and presentation but the longest interval to intervention. Reasons for delayed surgery varied, including patient desire to forego surgery, delayed radiologic diagnosis, and lack of surgical resources or personnel. 19% of patients in Group D had previous sternotomy, but there was no evidence to suggest that this factor led to either additional preoperative studies or delay in surgery. It is possible that adhesions from prior sternotomy resulted in lower likelihood of aortic rupture or tamponade, and thus lower mortality, but there was no statistical data to corroborate this. One explanation for low Group D mortality could be that by virtue of simply having survived a longer pre-operative interval, these patients self-selected for high likelihood of survival. Davies and colleagues reported that 47% of patients in their series presented 48 h or longer after symptom onset, and that these patients underwent surgery an average of 8 days after symptom onset with postoperative survival similar to those who presented early and underwent immediate treatment^21^. While we do not advocate for delayed surgery in ATAAD, our results also imply that a subset of patients who survive the initial 12 h after ATAAD diagnosis and remain clinically stable might successfully undergo delayed surgery without compromising outcomes.

As a tertiary referral and designated aortic center for the state, our institution receives ATAAD patients in transfer from other facilities located up to 200 miles away. In many cases, our institution is not geographically the closest cardiac surgery center from the referring facility. Our practice has been to recommend emergent surgery, often with admission directly to the OR. Since medical transportation, whether by ground or air ambulance, can take several hours, we were interested in examining the effect of this time interval on surgical outcomes. We hypothesized that the TtI would be affected by the distance between our institution and the referring facility. However, our data do not demonstrate any significant relationship between transfer distance and outcomes. Our results are similar to those from other studies, which have demonstrated that interhospital transfer does not correlate with time to treatment or outcomes^22,23^.

Our results present some key implications: (1) the importance of early presentation after symptom onset; (2) the importance of expedited treatment once the ATAAD is diagnosed; and (3) the question of optimal destination for ATAAD treatment based on TtI. Should time of symptom onset and anticipated TtI play a role in determining destination for transfer? If transfer to a tertiary or aortic center would result in a TtI greater than 4 h while transfer to a closer non-tertiary center would result in TtI less than 4 h, could the latter be the better choice? While our data suggests that transfer distance itself is not correlated with time to treatment, rapid intervention is a key factor affecting mortality especially as TtI approaches 8 h. Dedicated aortic centers are relatively rare and often located in major urban centers. Furthermore, all cardiac surgeons at an aortic center are not necessarily aortic subspecialists. On the other hand, many cardiac surgeons in community settings safely perform aortic surgery, including treatment of ATAAD. Thus, it could be argued that if the anticipated TtI is high, the most appropriate strategy may be to transfer to the closest hospital offering aortic surgical services. One question for further study could be whether there is benefit of seeking care at a dedicated aortic center, even if such a center is geographically significant farther away than a closer hospital with surgical staff capable to treating ATAAD.

Several institutions have implemented systematic processes such as transfer protocols to improve and expedite care of such critically ill patients^27,28^. Our institution has a “Level 1” protocol to activate necessary personnel and resources (e.g., cardiac surgery, anesthesia, critical care, operating room staff, nursing) to facilitate acceptance and care of ATAAD patients. Part of this protocol is creating a method whereby referring hospitals can directly upload any radiologic images to an online server so that the receiving physician can view them even before speaking with the referring physician through the Transfer Center. Use of technology such as this can reduce any delays in treatment. However, even with such protocols, often the fact that the referring and receiving institutions are not affiliated (many times are part of different health systems) can creates hurdles. Often, the mode of transport and arranging physical transportation of the patient is dictated by the referring institution and affected by resource availability.

This study is subject to the inherent limitations of its retrospective, single-center design. A prospective study examining the effect of TtI would be difficult, if not impossible, to conduct given existing practice standards and expectations of immediate surgical treatment for ATAAD. Our results could also be affected by the distribution and low sample sizes among subgroups. An additional limitation is that our study utilizes a subjective reporting of symptom onset and reliance on accuracy of ED medical record documentation. We elected to utilize the time stamp on the imaging study as a standard objective proxy for time of diagnosis as we believe that this measure provides a definitive timepoint for analysis. In addition, our medical records did not include method of transfer (air vs. ground), which is data that could help delineate effects on TtI and suggest potential areas for system-based practice change. Lastly, this study does not capture patients with ATAAD that died without undergoing surgery. This type of data, which is based on billing records at our institution and access restricted, can certainly provide valuable perspective regarding true mortality of ATAAD.

Our results demonstrate that surgical intervention within 4 h of diagnosis leads to significantly better outcomes; ATAAD must continue to be considered a time-sensitive emergency and steps taken to expedite surgery. In ATAAD patients requiring transfer, time-dependent outcomes should be considered when determining the optimal treatment strategy, and transportation time and expected time to treatment should be factored into deciding the destination for these patients.

## References

[1] Coady MA, Rizzo JA, Goldstein LJ, Elefteriades JA (1999). Natural history, pathogenesis, and etiology of thoracic aortic aneurysms and dissections. Cardiol. Clin..

[2] Kurz SD (2017). Insight into the incident of acute aortic dissection in the German region of Berlin and Brandenburg. Int. J. Card..

[3] Hagan PG (2000). The international registry of acute aortic dissection (IRAD): new insights into an old disease. JAMA.

[4] Meszaros I (2000). Epidemiology and clinicopathology of aortic dissection. Chest.

[5] Moon MR (2009). Approach to the treatment of aortic dissection. Surg. Clin. N. Am..

[6] Hirst AE, Johns VJ, Kime SW (1958). Dissecting aneurysm of the aorta: a review of 505 cases. Medicine (Baltimore).

[7] Evangelista A (2018). Insights from the international registry of acute aortic dissection: a 20-year experience of collaborative clinical research. Circulation.

[8] Rampoldi V (2007). Simple risk models to predict surgical mortality in acute type a aortic dissection: the international registry of acute aortic dissection score. Ann. Thorac. Surg..

[9] Tsai TT (2006). Long-term survival in patients presenting with type a acute aortic dissection: insights from the international registry of acute aortic dissection (IRAD). Circulation.

[10] Bonser RS (2011). Evidence, lack of evidence, controversy, and debate in the provision and performance of the surgery of acute type a aortic dissection. J. Am. Coll. Cardiol..

[11] Mehta RH (2002). Predicting death in patients with acute type a aortic dissection. Circulation.

[12] Olsson C, Thelin S, Stahle E, Ekbom A, Granath F (2006). Thoracic aortic aneurysm and dissection: increasing prevalence and improved outcomes reported in a nationwide population-based study of more than 14,000 cases from 1987 to 2002. Circulation.

[13] Olsson C (2017). Medium-term survival after surgery for acute type a aortic dissection is improving. Eur. J. Cardiothorac. Surg..

[14] Yuan X, Mitsis A, Tang Y, Nienaber CA (2019). The IRAD and beyond: what have we unravelled so far?. Gen. Thorac. Cardiovasc. Surg..

[15] Parikh N (2017). Changes in operative strategy for patients enrolled in the international registry of acute aortic dissection interventional cohort program. J. Thorac. Cardiovasc. Surg..

[16] Rapezzi C (2008). Risk factors for diagnostic delay in acute aortic dissection. Am. J. Cardiol..

[17] Lee TC (2018). Contemporary management and outcomes of acute type a aortic dissection: an analysis of the STS adult cardiac surgery database. J. Card. Surg..

[18] Fan PY (2019). Impact of renal dysfunction on surgical outcomes in patients with aortic dissection. Medicine (Baltimore).

[19] Berretta P (2018). Malperfusion syndromes in type a aortic dissection: what we have learned from IRAD. J. Vis. Surg..

[20] Rice RD (2015). Is total arch replacement associated with worse outcomes during repair of acute type a aortic dissection?. Ann. Thorac. Surg..

[21] Davies RR (2007). Thoracic surgery directors association award. What is the optimal management of late-presenting survivors of acute type a aortic dissection?. Ann. Thorac. Surg..

[22] Tseng YH (2019). Does interhospital transfer influence the outcomes of patients receiving surgery for acute type a aortic dissection? Type a aortic dissection: is transfer hazardous or beneficial?. Emerg. Med. Int..

[23] Matschilles, C., Mochmann, H., Syrmas, G., Zaschke, L. & Kurz, S. *Interhospital transfer of patients suffering from acute aortic dissection by helicopter and ground-based emergency medical services*. European Resuscitation Council (ERC) Congress. 10.26226/morressier.5b51cf46b1b87b0009beb6c1 (2018).

[24] Harris KM (2011). Correlates of delayed recognition and treatment of acute type a aortic dissection: the international registry of acute aortic dissection (IRAD). Circulation.

[25] Froehlich W (2018). Delay from diagnosis to surgery in transferred type a aortic dissection. Am. J. Med..

[26] Zaschke L (2020). Acute type a aortic dissection: aortic dissection detection risk score in emergency care-surgical delay because of initial misdiagnosis. Eur. Heart J. Acute Cardiovasc. Care.

[27] Harris KM (2010). Multidisciplinary standardized care for acute aortic dissection: design and initial outcomes of a regional care model. Circ. Cardiovasc. Qual. Outcomes.

[28] Andersen ND (2014). Outcomes of acute type a dissection repair before and after implementation of a multidisciplinary thoracic aortic surgery program. J. Am. Coll. Cardiol..

